# Etoposide dosing challenges in a testicular cancer patient with hepatic impairment and drug-drug interactions–a case report

**DOI:** 10.1007/s00280-026-04865-1

**Published:** 2026-02-03

**Authors:** Catharina J. P. Op ‘t Hoog, Loek A. W. de Jong, Joost Sijm, Minke Smits, Sasja F. Mulder, Emmy Boerrigter

**Affiliations:** 1 Department of Pharmacy, Pharmacology & Toxicology, Radboud University Medical Center, P.O. box 9101, Nijmegen, 6525 GA The Netherlands; 2 Department of Medical Oncology, Radboud University Medical Center, Nijmegen, 6525 GA The Netherlands

**Keywords:** Testicular cancer, Seminoma, Cystic fibrosis, Liver cirrhosis, Drug-drug interactions, Etoposide

## Abstract

**Purpose:**

Etoposide, together with cisplatin, is a cornerstone in the treatment of metastatic testicular cancer, but dosing can be challenging in patients with organ dysfunction and/or polypharmacy. We report a patient with cystic fibrosis, liver cirrhosis and preexisting pancytopenia grade 1, requiring multiple concomitant medications inhibiting CYP3A4 and P-gp, who presented with metastatic seminoma.

**Method and results:**

Due to potential drug-drug interactions and liver cirrhosis, the initial etoposide dose was reduced to 50% of standard. Monitoring of etoposide plasma concentrations showed that total exposure was not enhanced. The subsequent dose was increased to 100% and further adjusted based on tolerability. After a dose reduction from cycle 2 due to pancytopenia grade 4, the patient successfully completed the chemotherapy regimen and had a complete response on first evaluation.

**Conclusion:**

This case shows that monitoring of etoposide plasma concentrations can be beneficial in complex clinical scenarios involving organ dysfunction and/or potential drug-drug interactions. This is especially important in curative treatment to avoid under dosing.

**Patient summary:**

This case report highlights the challenges of dosing etoposide in a patient with cystic fibrosis and liver cirrhosis who is taking multiple drugs that may interact with etoposide. The patient should be monitored closely on how the treatment is tolerated and the dose should be adjusted accordingly.

## Background

Testicular cancer is the most common type of cancer in males between the age of 14 to 40 years [1]. Staging and risk classification of testicular cancer is relevant for prognosis and treatment decisions, and are carried out according to the International Germ Cell Cancer Collaborative Group (IGCCCG) [2]. Stage one seminoma tumors are mainly treated with orchidectomy, with over 85% chance of cure [1, 3]. Lymph nodes are the main site for metastasis, and are mainly treated with radiotherapy or chemotherapy in stage IIA/B [1, 3]. Even with metastatic disease, chance of cure is still over 85% [1, 3]. Treatment with either three cycles of bleomycin, etoposide and cisplatin (BEP) or four cycles of etoposide and cisplatin (EP) are the recommended regimen in good risk metastatic seminoma tumors [1, 3, 4].

In these treatment regimens, etoposide, together with cisplatin, plays a central role, but its dosing can be challenging in patients with renal or hepatic impairment, or in patients with polypharmacy. Etoposide clearance occurs via both renal excretion and by hepatic metabolism. Approximately one-third of the drug is excreted unchanged in urine, while the rest is either excreted in the bile or undergoes hepatic metabolism into both active and inactive metabolites [5–7]. Hepatic metabolism is primarily mediated by the cytochrome P450 enzyme 3A4 (CYP3A4) [8]. The CYP3A4 mediated O-demethylated metabolites (catechol and quinone) are thought to have similar potency as etoposide [9]. Although these metabolites are less abundant in the plasma than etoposide, they are thought to contribute to both the efficacy and toxicity of etoposide [10, 11]. Additionally, etoposide is a substrate of the drug efflux transporter P-glycoprotein (P-gp) [12]. Consequently, concomitant use of CYP3A4 and P-gp inhibitors or inducers can significantly alter etoposide exposure [12]. For example, etoposide exposure was increased by 20% in combination with the CYP3A4 inhibitor ketoconazole, while etoposide clearance was increased 2-fold in combination with the CYP3A4 inducer mitotane, resulting in a decreased exposure [13, 14] Additionally, co-administration with the weak CYP3A4 and strong P-gp inhibitor cyclosporin has been shown to increase etoposide exposure by 50% to 80%, leading to a recommended dose reduction of 50% [15]. Etoposide dosing is further complicated in organ dysfunction. While the drug label provides clear recommendations for dose reduction in patients with renal impairment, it does not mandate dose adjustments for hepatic impairment. Nevertheless, previous studies suggest that elevated bilirubin levels and hypoalbuminemia correlate with increased unbound etoposide concentrations, resulting in increased toxicity, such as myelosuppression [16–18]. Therefore, a dose reduction of 50% may be considered in patients with high bilirubin or low albumin levels [16, 19].

Taken together, these factors highlight the challenges of etoposide dosing in patients with polypharmacy and organ dysfunction. In this case report, we describe a patient with advanced liver cirrhosis and cystic fibrosis (CF), requiring treatment with drugs that inhibit both CYP3A4 and P-gp, who presented with metastatic testicular cancer requiring systemic treatment with an etoposide-based chemotherapy regimen.

## Case presentation

A 27-year-old Caucasian male was initially diagnosed with stage one good risk seminoma of the testis at the age of 26, which was initially managed with a left inguinal total orchidectomy. He has CF (homozygous F508del), complicated with multiple hospital admissions, liver cirrhosis (Child-Pugh B), pancreatic insufficiency and preexisting pancytopenia grade 1. The patient was diagnosed with lymph node metastasis 2 months after surgery, for which he received proton radiotherapy. Due to a relapse 8 months after radiotherapy, EP-chemotherapy was recommended in accordance with the guidelines [1, 3]. Bleomycin was omitted to avoid further pulmonary toxicity in the context of his CF. Baseline laboratory values (with their reference values) before start of chemotherapy were: hemoglobin (Hb) 6.8 mmol/L (8.4–10.8 mmol/L); leukocytes 3.2*10^9^/L (4.0–10.0*10^9^/L); neutrophils 1.23*10^9^/L (1.50–7.50*10^9^/L); thrombocytes 103*10^9^/L (150–400*10^9^/L); international normalized ratio (INR) 1.4 (0.8–1.2); creatinine 97 µmol/L (60–110 µmol/L); estimated glomerular filtration rate (eGFR) CKD-EPI > 90 ml/min/1.73 m^2^ (> 90 ml/min/1.73 m^2^); alanine aminotransferase (ALAT) 17 U/L (< 45 U/L); aspartate aminotransferase (ASAT) 37 U/L (< 35 U/L); lactate dehydrogenase (LDH) 270 U/L (< 250 U/L); alkaline phosphatase 135 U/L (< 115 U/L); bilirubin total 20 µmol/L (< 17 µmol/L) and conjugated 17 µmol/L (< 5 µmol/L); albumin 24 g/L (35–50 g/L). Concomitant drugs and supplements used by the patient before start of chemotherapy can be found in Table 1. Before start of chemotherapy, the clinical pharmacist was consulted for possible drug-drug interactions, which were checked by the automated warnings in the prescribing system and with the use of UpToDate Lexidrug database [20]. For his CF, the patient used ivacaftor/tezacaftor/elexacaftor, of which ivacaftor is a weak P-gp and CYP3A4 inhibitor [21], and azithromycin prophylaxis therapy, which is a weak P-gp inhibitor [22]. Since etoposide is metabolized by CYP3A4 and is a substrate for P-gp, co-administration of these drugs may lead to increased exposure and consequently increased toxicity [12]. Additionally, due to his liver cirrhosis, the patient had low albumin levels, which could result in higher unbound etoposide concentrations and an increased risk of toxicity [16, 18].

**Table 1:** Overview of drugs and supplements used by the patient before start of chemotherapy

Drug name	Dose
Pancreatin 300 mg (25,000 units lipase/18,000 units amylase/1000 units protease)	8–9 times per day, when needed
Azithromycin tablets	500 mg 3 times per week
Beclomethasone/formoterol inhalation 100/6 µg/dose	Once daily, when needed
Carvedilol tablets	6.25 mg once daily
Cholecalciferol (vitamin D) tablets	800 IE once daily
Cyanocobalamin tablets	1000 µg once daily
Multivitamins/minerals (with ADEK, folate, iron) capsules	Twice daily
Ivacaftor tablets	150 mg once daily
Ivacaftor/tezacaftor/elexacaftor tablets	75/50/100 mg once daily
Macrogol/electrolytes sachets	When needed
NaCl (sodium chloride) 5.85% inhalation	4 mL twice daily
Pantoprazole tablets	40 mg once daily
Salbutamol inhalation 200 µg/dose	4 times per day, when needed
Ursodeoxycholic acid tablets	450 mg twice daily

**Fig. 1:**
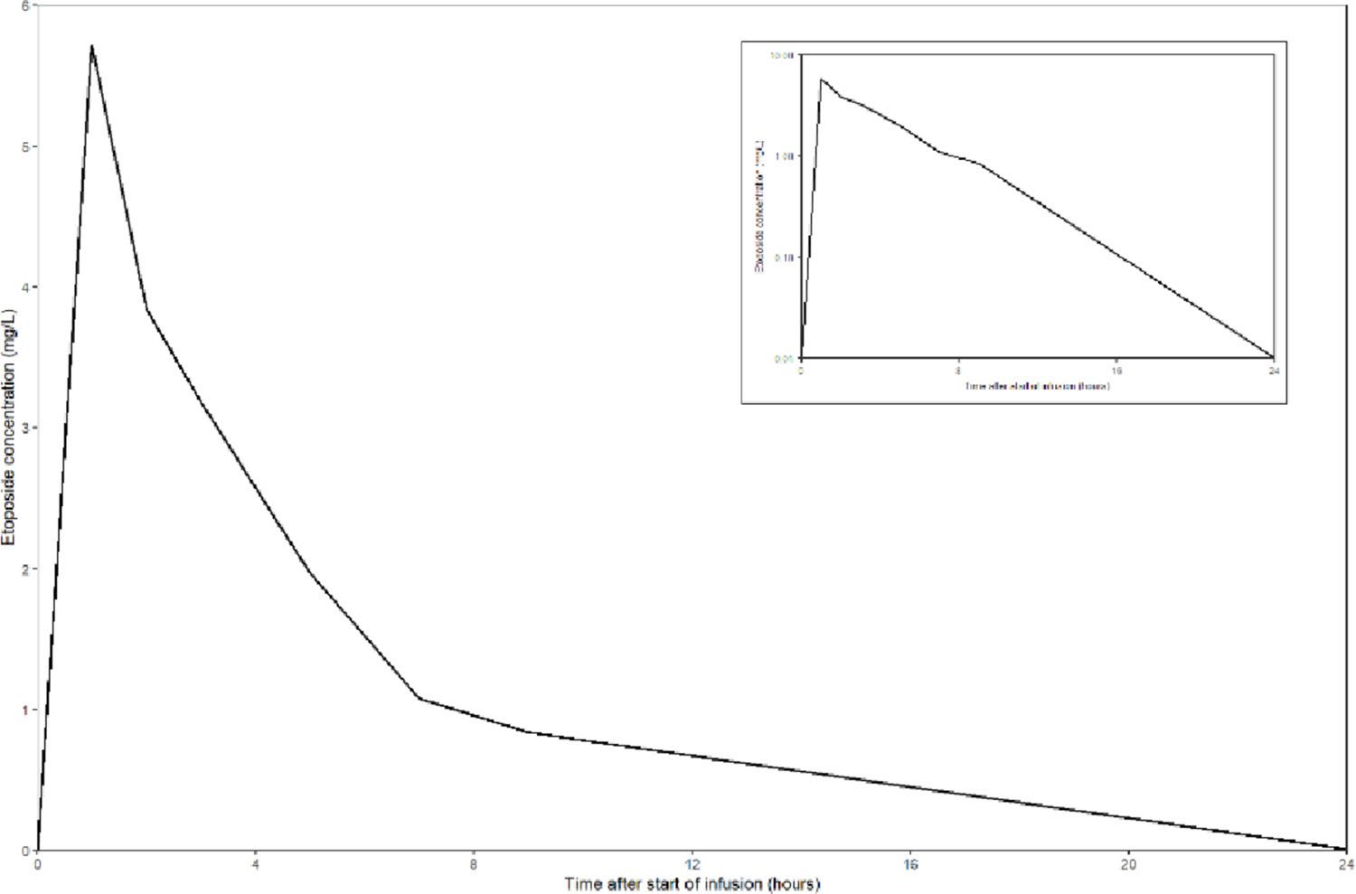
Plasma concentration time-curve of etoposide following intravenous administration of 50 mg/m^2^. Inset graph is plotted on a semi-log scale

To mitigate this risk, the initial dose of the first cycle was reduced to 50 mg/m^2^ (50% of the normal dose) and was administered with an infusion duration of one hour. Additionally, the patient received daily intravenous albumin suppletion during the cycle in combination with spironolactone to prevent ascites due to the high infusion volume (4.5 L per day). He received vitamin K suppletion to normalise his INR and granulocyte-colony stimulating factor (G-CSF) to support his bone marrow. Pharmacokinetic sampling was performed after the first dose. Plasma concentrations were analyzed using a validated ultra-performance liquid chromatography with fluorescence detection method [23]. The calculated 24-hour total exposure (area under the curve; AUC) was 28 mg*h/L (Table 2; Fig. 1). In comparison, the etoposide exposure at the normal dose of 100 mg/m^2^ is approximately 85 mg*h/L with an interpatient variability of ~ 28% [23–25]. Correcting for hypoalbuminemia, the unbound fraction of etoposide was approximately 6% versus 4% in patients with normal albumin [26]. The unbound concentration of a drug represents the pharmacologically active component. Based on an estimated unbound fraction of 6% in this patient due to hypoalbuminemia, the predicted unbound AUC is 1.68 mg*h/L, corresponding to roughly 50% of the expected unbound exposure at the standard dose of ~ 3.4 mg*h/L. Since the observed exposure was lower than expected and corresponded to the 50% dose reduction in this patient, we concluded that the initial concern regarding excessive exposure due to the drug-drug interactions and liver cirrhosis was not warranted. As a result, the dose was increased to 100 mg/m^2^ (100%) on day 3 to 5 of the first cycle to restore the expected exposure. The absolute infused dose of etoposide of the first cycle was 400 mg/m^2^ (normally 500 mg/m^2^ per cycle is given). Due to a pancytopenia grade 4 (Hb 5.3 mmol/L, neutrophils 0.0*10^9^/L and thrombocytes 26*10^9^/L) with hematuria and neutropenic fever, for which he was admitted to the hospital and treated with antibiotics, the etoposide dose was adjusted to 75% for cycles 2 to 4. The additional etoposide cycles were well tolerated, and the patient completed his chemotherapy regimen successfully. The patient showed a complete response, with no signs of recurrence 5 months after treatment completion.

**Table 2:** Measured plasma Etoposide concentrations following the 50 mg/m^2^ dose in cycle 1

Sample time	Etoposide total plasma concentration (mg/L)
Immediately after the end of the infusion	5.71
1 h after the end of the infusion	3.84
2 h after the end of the infusion	3.18
4 h after the end of the infusion	1.97
6 h after the end of the infusion	1.08
8 h after the end of the infusion	0.84
24 h after the end of the infusion	0.01

## Discussion

Our case highlights the challenges of etoposide dosing in the context of both concomitant drug-drug interactions and organ dysfunction. In our patient, a 50% dose reduction was considered the optimal starting dose, taking into account the potential influence of CYP3A4 and P-gp inhibitors, as well as his hepatic impairment. Despite potential drug-drug interactions and the presence of advanced liver cirrhosis, the effect on etoposide exposure appeared to be limited. This underscores that the impact of drug-drug interactions and hepatic dysfunction on etoposide clearance is unpredictable, and thus preemptive dose reductions should be made with caution. Close monitoring and dose adjustment based on tolerability remain essential to avoid the risk of underdosing, especially in the curative setting.

Drug-drug interactions warrant careful evaluation. Earlier research suggests that the effect of CYP3A4 inhibition on etoposide exposure is minor [23]. In contrast, previous studies researching the effect of strong CYP3A4 inhibitors or inducers showed varying effects on etoposide exposure [13, 14]. Additionally, the effect of weak or moderate P-gp inhibitors on etoposide exposure seems negligible [23, 27]. Similarly, in our case no increase in total etoposide exposure was observed when combined with weak CYP3A4 and P-gp inhibitors. Notably, only cyclosporin and mitotane had a clinically relevant effect on etoposide exposure in earlier research through P-gp inhibition and CYP3A4 induction, respectively [13, 15, 28]. Information on the clinical relevance of the effect on etoposide efficacy and toxicity is scarce. Therefore, drug-drug interaction data cannot be broadly extrapolated for etoposide and dose modifications should be considered carefully and guided by clinical and/or pharmacokinetic monitoring.

In patients with hepatic impairment, elevated bilirubin is a marker for liver function, specifically for reduced hepatic clearance and synthesis. This can lead to low albumin levels, resulting in increased unbound etoposide concentrations, which increases the toxicity risk [18, 29]. Additionally, renal impairment can significantly increase etoposide exposure [17]. In our patient, renal function was adequate and bilirubin was slightly increased. Although we were unable to measure the unbound etoposide concentration, the low albumin level allowed estimation of the unbound fraction. The albumin-adjusted predicted exposure in our case was consistent with literature values when corrected for the 50% starting dose [23, 25]. This implies that the total exposure was not affected by either drug-drug interactions or hepatic impairment. However, it remains uncertain whether the exposure would have been even lower in the absence of the drug-drug interactions or hepatic impairment. Although the etoposide dose was increased to 100% after measurement of the etoposide plasma concentrations, further dose reduction to 75% for cycle 2 to 4 were warranted in our patients. It is unclear if this was due to an elevated unbound etoposide concentration or other factors. Further plasma concentrations were not measured. In more complex scenarios, particularly when both albumin is low and bilirubin is elevated, measurement of unbound etoposide would be more informative. Some reports recommend a 50% initial dose reduction in patients with hepatic impairment, specifically in the case of high bilirubin and low albumin levels [16, 18, 19]. However, as highlighted in our case, close monitoring of the patient is needed in order to further adjust the dose based on tolerability.

In conclusion, the presented case supports that the impact of drug-drug interactions and hepatic impairment on etoposide exposure is unpredictable. Although monitoring of etoposide concentrations is not routine in clinical practice, our case highlights its potential value in complex clinical scenarios involving drug-drug interactions or organ dysfunction. When only total etoposide plasma concentrations are available, the effect of albumin concentrations and renal function should be taken into account to estimate the unbound etoposide exposure. Etoposide dosing should not be guided by a single parameter, but rather by the combined effect of these parameters to individualize the dose to prevent toxicity and optimize efficacy. Preemptive dose reductions should be made with caution, with close monitoring and adjustment of the dose based on tolerability to avoid the risk of underdosing in this curative setting.

## Data Availability

Data are available on reasonable request from the authors.

## References

[1] Oldenburg J, Berney DM, Bokemeyer C, Climent MA, Daugaard G, Gietema JA et al (2022) Testicular seminoma and non-seminoma: ESMO-EURACAN clinical practice guideline for diagnosis, treatment and follow-up. Ann Oncol 33(4):362–75 35065204 10.1016/j.annonc.2022.01.002

[2] Beyer J, Collette L, Sauve N, Daugaard G, Feldman DR, Tandstad T et al (2021) Survival and new prognosticators in metastatic seminoma: results from the IGCCCG-update consortium. J Clin Oncol 39(14):1553–62 33729863 10.1200/JCO.20.03292 PMC8099394

[3] European Association of Urology (EAU) (2025) EAU Guidelines on Testicular Cancer. European Association of Urology

[4] Raggi D, Chakrabarti D, Cazzaniga W, Aslam R, Miletic M, Gilson C et al (2025) Management of Testicular Cancer. JCO Oncology Practice 10.1200/OP-25-00211 40408609

[5] Hande KR (1998) Etoposide: four decades of development of a topoisomerase II inhibitor. Eur J Cancer 34(10):1514–1521 9893622 10.1016/s0959-8049(98)00228-7

[6] Slevin ML (1991) The clinical pharmacology of etoposide. Cancer 67(S1):319–329 1984835 10.1002/1097-0142(19910101)67:1+<319::aid-cncr2820671319>3.0.co;2-d

[7] Creaven PJ (1982) The clinical pharmacology of VM26 and VP16-213. Cancer Chemother Pharmacol 7(2):133–140 7044592 10.1007/BF00254535

[8] Relling MV, Nemec J, Schuetz EG, Schuetz JD, Gonzalez FJ, Korzekwa KR (1994) O-demethylation of epipodophyllotoxins is catalyzed by human cytochrome P450 3A4. Mol Pharmacol 45(2):352–358 8114683

[9] Lovett BD, Strumberg D, Blair IA, Pang S, Burden DA, Megonigal MD et al (2001) Etoposide metabolites enhance DNA topoisomerase II cleavage near leukemia-associated MLL translocation breakpoints. Biochemistry 40(5):1159–70 11170441 10.1021/bi002361x

[10] Stremetzne S, Jaehde U, Kasper R, Beyer J, Siegert W, Schunack W (1997) Considerable plasma levels of a cytotoxic etoposide metabolite in patients undergoing high-dose chemotherapy. Eur J Cancer 33(6):978–979 9291827 10.1016/s0959-8049(97)00087-7

[11] Cai X, Woo MH, Edick MJ, Relling MV (1999) Simultaneous quantitation of etoposide and its catechol metabolite in human plasma using high-performance liquid chromatography with electrochemical detection. J Chromatogr B Biomed Sci 728(2):241–250 10.1016/s0378-4347(99)00110-3 10406209

[12] Hikma Pharmaceuticals (2024) Summary of Product Characteristics Etoposide

[13] Jouinot A, Royer B, Chatelut E, Moeung S, Assie G, Thomas-Schoemann A et al (2018) Pharmacokinetic interaction between mitotane and etoposide in adrenal carcinoma: a pilot study. Endocr Connect 7(12):1409–14 30533000 10.1530/EC-18-0428 PMC6301193

[14] Yong WP, Desai AA, Innocenti F, Ramirez J, Shepard D, Kobayashi K et al (2007) Pharmacokinetic modulation of oral etoposide by ketoconazole in patients with advanced cancer. Cancer Chemother Pharmacol 60(6):811–9 17308893 10.1007/s00280-007-0428-5

[15] Lum BL, Kaubisch S, Yahanda AM, Adler KM, Jew L, Ehsan MN et al (1992) Alteration of etoposide pharmacokinetics and pharmacodynamics by cyclosporine in a phase I trial to modulate multidrug resistance. J Clin Oncol 10(10):1635–42 1403041 10.1200/JCO.1992.10.10.1635

[16] Stewart CF, Arbuck SG, Fleming RA, Evans WE (1990) Changes in the clearance of total and unbound etoposide in patients with liver dysfunction. J Clin Oncol 8(11):1874–1879 2230875 10.1200/JCO.1990.8.11.1874

[17] Stewart CF (1994) Use of Etoposide in patients with organ dysfunction: Pharmacokinetic and pharmacodynamic considerations. Cancer Chemother Pharmacol 34(Suppl):S76–83 8070032 10.1007/BF00684868

[18] Stewart CF, Arbuck SG, Fleming RA, Evans WE (1991) Relation of systemic exposure to unbound etoposide and hematologic toxicity. Clin Pharmacol Ther 50(4):385–393 1914374 10.1038/clpt.1991.155

[19] Giraud EL, de Lijster B, Krens SD, Desar IME, Boerrigter E, van Erp NP (2023) Dose recommendations for anticancer drugs in patients with renal or hepatic impairment: an update. Lancet Oncol 24(6):e229 37269847 10.1016/S1470-2045(23)00216-4

[20] UpToDate Lexidrug Interactions: UpToDate Inc.; [6 February 2025]. Available from: https://online.lexi.com/lco/action/interact

[21] Vertex Pharmaceuticals (2022) (Ireland) Limited. Summary of Product Characteristics Kalydeco

[22] Pfizer (2017) Summary of Product Characteristics Zythromax

[23] Strik J, de Jong LAW, Sijm J, Desar IME, van Erp NP (2024) Effect of aprepitant on etoposide pharmacokinetics in patients with testicular cancer: a pharmacokinetic study to determine the absence of a clinically relevant interaction. Clin Pharmacol Ther 115(1):135–8 37867292 10.1002/cpt.3081

[24] Hande K, Messenger M, Wagner J, Krozely M, Kaul S (1999) Inter- and intrapatient variability in etoposide kinetics with oral and intravenous drug administration. Clin Cancer Res 5(10):2742–2747 10537337

[25] Thomas HD, Porter DJ, Bartelink I, Nobbs JR, Cole M, Elliott S et al (2002) Randomized cross-over clinical trial to study potential pharmacokinetic interactions between cisplatin or carboplatin and etoposide. Br J Clin Pharmacol 53(1):83–91 11849199 10.1046/j.0306-5251.2001.01513.x PMC1874557

[26] Stewart CF, Pieper JA, Arbuck SG, Evans WE (1989) Altered protein binding of etoposide in patients with cancer. Clin Pharmacol Ther 45(1):49–55 2910637 10.1038/clpt.1989.8

[27] van de Poll ME, Relling MV, Schuetz EG, Harrison PL, Hughes W, Flynn PM (2001) The effect of atovaquone on etoposide pharmacokinetics in children with acute lymphoblastic leukemia. Cancer Chemother Pharmacol 47(6):467–72 11459198 10.1007/s002800000250

[28] Bisogno G, Cowie F, Boddy A, Thomas HD, Dick G, Pinkerton CR (1998) High-dose cyclosporin with etoposide–toxicity and pharmacokinetic interaction in children with solid tumours. Br J Cancer 77(12):2304–2309 9649150 10.1038/bjc.1998.383 PMC2150390

[29] Joel SP, Shah R, Clark PI, Slevin ML (1996) Predicting etoposide toxicity: relationship to organ function and protein binding. J Clin Oncol 14(1):257–267 8558207 10.1200/JCO.1996.14.1.257

